# Changes in Vascular Permeability and Expression of Different Angiogenic Factors Following Anti-Angiogenic Treatment in Rat Glioma

**DOI:** 10.1371/journal.pone.0008727

**Published:** 2010-01-15

**Authors:** Meser M. Ali, Branislava Janic, Abbas Babajani-Feremi, Nadimpalli R. S. Varma, A. S. M. Iskander, John Anagli, Ali S. Arbab

**Affiliations:** Cellular and Molecular Imaging Laboratory, Department of Radiology, Henry Ford Hospital, Detroit, Michigan, United States of America; Genentech, United States of America

## Abstract

**Background:**

Anti-angiogenic treatments of malignant tumors targeting vascular endothelial growth factor receptors (VEGFR) tyrosine kinase are being used in different early stages of clinical trials. Very recently, VEGFR tyrosine kinase inhibitor (Vetanalib, PTK787) was used in glioma patient in conjunction with chemotherapy and radiotherapy. However, changes in the tumor size, tumor vascular permeability, vascular density, expression of VEGFR2 and other angiogenic factors in response to PTK787 are not well documented. This study was to determine the changes in tumor size, vascular permeability, fractional plasma volume and expression of VEGFR2 in PTK787 treated U-251 glioma rat model by *in vivo* magnetic resonance imaging (MRI) and single photon emission computed tomography (SPECT). The findings were validated with histochemical and western blot studies.

**Methodologies and Principal Findings:**

Seven days after implantation of U251 glioma cells, animals were treated with either PTK787 or vehicle-only for two weeks, and then tumor size, tumor vascular permeability transfer constant (K^trans^), fractional plasma volume (fPV) and expression of VEGFR2 and other relevant angiogenic factors were assessed by *in vivo* MRI and SPECT (Tc-99-HYNIC-VEGF), and by immunohistochemistry and western blot analysis. Dynamic contrast-enhanced MRI (DCE-MRI) using a high molecular weight contrast agent albumin-(GdDTPA) showed significantly increased K^trans^ at the rim of the treated tumors compared to that of the central part of the treated as well as the untreated (vehicle treated) tumors. Size of the tumors was also increased in the treated group. Expression of VEGFR2 detected by Tc-99m-HYNIC-VEGF SPECT also showed significantly increased activity in the treated tumors. In PTK787-treated tumors, histological staining revealed increase in microvessel density in the close proximity to the tumor border. Western blot analysis indicated increased expression of VEGF, SDF-1, HIF-1α, VEGFR2, VEGFR3 and EGFR at the peripheral part of the treated tumors compared to that of central part of the treated tumors. Similar expression patters were not observed in vehicle treated tumors.

**Conclusion:**

These findings indicate that PTK787 treatment induced over expression of VEGF as well as the Flk-1/VEGFR2 receptor tyrosine kinase, especially at the rim of the tumor, as proven by DCE-MRI, SPECT imaging, immunohistochemistry and western blot.

## Introduction

Malignant gliomas are among the most devastating tumors, with survival of only one to three years after diagnosis, even with the best of treatments combining surgery, radiation and chemotherapy [1], [2]. Because of the hypervascular nature of glioblastoma and the associated active angiogenesis, investigators have added anti-angiogenic treatment as an adjuvant to normalize blood vessels and control abnormal angiogenesis [3], [4], [5], [6]. Angiogenesis is essential for glioma tumor growth and metastasis. Malignant glioma cells release vascular endothelial growth factor (VEGF), an important regulator and promoter of angiogenesis [4]. Animal studies have indicated that angiogenesis and increased vascular permeability are essential for the proliferation and survival of glioma cells [7]. Vascular endothelial growth factor, also termed vascular permeability factor (VPF), is well-studied multifunctional cytokine considered to play a pivotal role in the induction of tumor angiogenesis. *In vitro* and *in vivo* data suggest that VEGF/VPF is an endothelial-cell specific mitogen [8]. In addition to having a mitogenic activity, VEGF/VPF is a potent vascular permeability enhancer [8]. VEGF/VPF has been shown to increase the permeability of micro vessels to plasma macromolecules with a potency approximating 50,000 times that of histamine [9]. Expression of VEGF and its receptors correlates to the degree of tumor vascularization and has been proposed as a prognostic factor for assessing patient survival [10]. High-affinity cognate VEGF endothelial receptors are VEGFR-1/Flt-1 and VEGFR-2/Flk-1/KDR and both receptors have been shown to be important regulatory factors for vasculogenesis and physiological angiogenesis [11]. However, the interaction of VEGF/VPF with Flk-1/VEGFR2 is thought to be more important for tumor angiogenesis because it is essential for induction of the full spectrum of VEGF/VPF functions [12]. In fact, many compounds and molecules developed to block VEGF/VPF activities mediated by Flk-1/VEGFR2 have been shown to have antiangiogenic activity in animal models [13], [14]. One such molecule is PTK787 that inhibits the phosphorylation of Flk-1/VEGFR2 and Flt-1 receptors by binding to and inhibiting the protein kinase domain of these receptors [15]. By directly inhibiting the phosphorylation of the VEGF/VPF receptor tyrosine kinases, PTK787 suppresses angiogenesis induced by VEGF/VPF. At slightly higher doses, it also inhibits PDGF receptor tyrosine kinase [15]. PTK787 demonstrated efficacy in preclinical and Phase I/II trials where it significantly reduced tumor vessel density and in some cases induced tumor regression [12]. PTK787 significantly inhibited growth of breast tumors *in vivo* and disrupted tumor microvasculature after five days of treatment [10]. However, it has also been noted that continued anti-angiogenic therapy targeting only the VEGF-VEGFR system might activate pro-angiogenic factors other than VEGF, such as basic fibroblast growth factor (bFGF), stromal derived factor 1 (SDF-1) and Tie2 [5], and may mobilize circulating endothelial cells and bone marrow derived precursor cells that are known to promote angiogenesis [5], [16], [17]. Thus, the inhibitory therapy targeting VEGF and/or VEGFRs may paradoxically end up enhancing angiogenic and pro-growth responses. Moreover, abundant VEGF over expression has been demonstrated in human malignant glioma animal model [4]. However, there has been no report on the changes in the expression pattern of different angiogenic factors in glioma following treatment with antiangiogenic agents.

Many pre-clinical and clinical studies have shown that Dynamic Contrast Enhancement (DCE) magnetic resonance imaging (MRI) can assess tumor perfusion to predict tumor angiogenesis and tumor response to anti-angiogenic therapy [18], [19]. This method monitors the pharmacokinetic uptake and wash-out of a MRI contrast agent within the extracellular space of tumor tissues that can be used to evaluate a vascular permeability coefficient (K^trans^), which has been shown to correlate with tumor perfusion and angiogenesis. Vascular permeability can be measured with a macromolecular contrast agents and small molecular agents; however these two agents are not equally sensitive to the cancerous tissues [20]. Macromolecular contrast agents are more sensitive to the differences in vascular permeability exhibited by drug treatment. Macromolecular contrast agent, albumin-(GdDTPA) have a spherical diameter of ∼6 nm and it appears to have the best balance between payload and size needed for DCE MRI studies [18], [19]. Therefore, albumin-(GdDTPA) has been used by many groups for MRI assessment of vascular permeability in experimental human glioma model. GdDTPA was used to detect and measure hypothesized changes in the tumor microvasculature induced by the blockade of VEGF receptors, as well as the accompanying changes in fractional plasma volume (fPV) and vascular permeability to the macromolecules. In an animal breast tumor model a high correlation between antiangiogenic therapy (bevacizumab) and response in the tumor vascular bed was demonstrated by using macromolecular contrast agent [21]. Availability of noninvasive assessment of vascular physiology within the tumor and its surrounding tissue would provide an extremely useful marker of response, and more critically, of non-response to antiangiogenic therapy.

Imaging modality such as single photon emission computed tomography (SPECT) can be used to analyze VEGF induced signaling pathways in response to antiangiogenic (PTK787) therapy. The effectiveness of treatment in rat glioma tumor vasculature can be easily assessed by monitoring a biomarker response (vascular surrogate) to PTK87 therapy. Since VEGFR2 is over expressed in tumor vasculature (40,000 copies/endothelial cell) and has a high binding affinity (K_a_ = 5 to 77×10^14^ M^−1^) to VEGF [22], VEGF receptors have been targeted as anti- and pro-angiogenic therapeutic agents as well as angiogenesis mediator based imaging agents [23], [24]. In an experimental breast cancer mouse model, VEGFR2 expression has been detected by Technetium-99m pertechnetate (Tc-99m) labeled VEGF [24]. Recently, site specific labeled Tc-99m-hydrazine nicotinamide (HYNIC)-VEGF has been used for the imaging of mouse model tumor (4T1 murine mammary carcinoma) vasculature before and after antiangiogenic therapies [22]. Backer et al. reported recombinant single-chain VEGF with a cysteine-containing tag that allows the site specific labeling of Tc-99m-HYNIC and other imaging agents [24]. All the studies showed that the accumulation of Tc-99m-HYNIC labeled VEGF derivatives was VEGFR2 receptor mediated. However, there has been no report on *in vivo* showing of the expression of VEGFR2 in glioma following treatment with antiangiogenic agent.

The purposes of this study were to determine: 1) whether antiangiogenic therapy using PTK787 effectively decreased the size of implanted glioma (U251) in a rat model, 2) the changes in expression of angiogenic factors in and around the tumors in response to PTK 787, 3) whether changes in vascular parameters could be determined *in vivo* by MRI and 4) whether changes in the expression of VEGF receptors could be determined by *in vivo* SPECT scanning. The findings were validated with immunohistochemistry and western blot studies.

## Results

### Tumor Size

**Figure 1:**
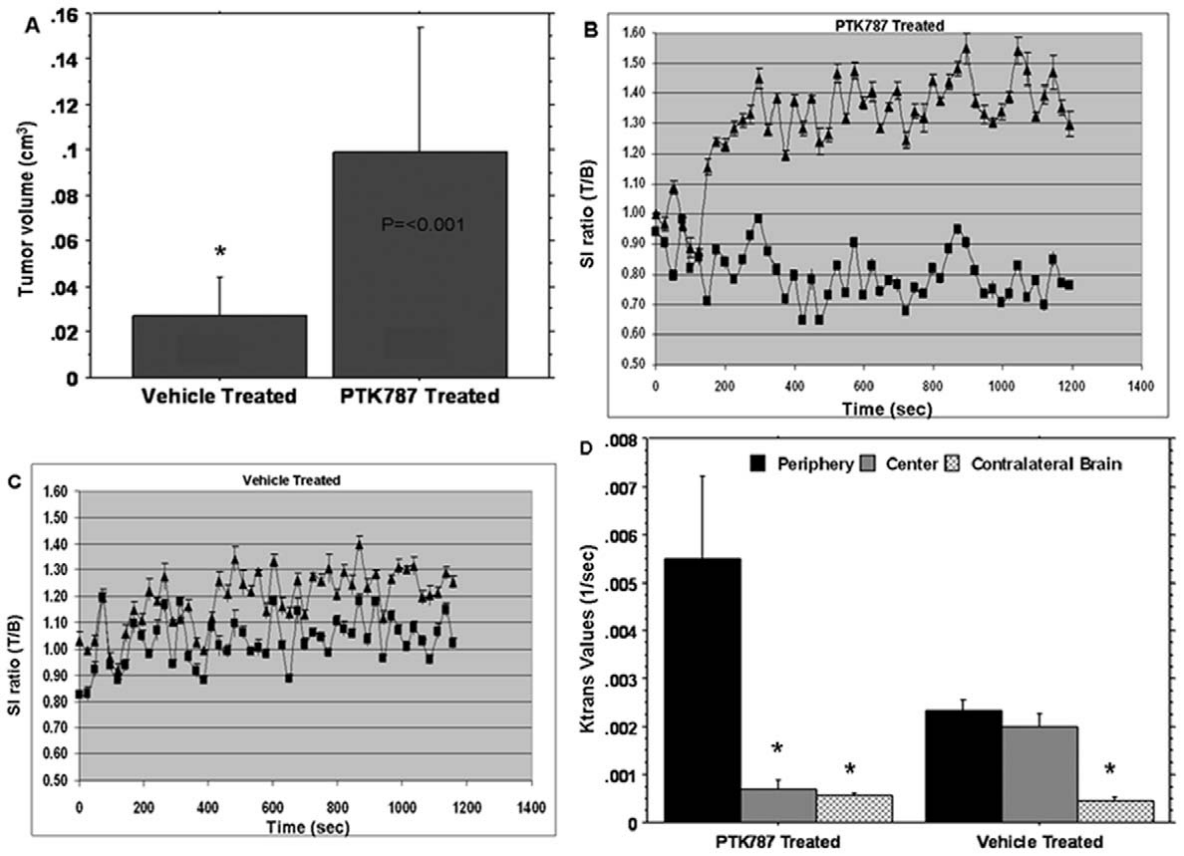
Tumor volume, signal intensity changes and K^trans^ in PTK787 and vehicle treated tumors. Measurement of tumor volume from the post contrast T1WI show significantly higher tumor volume in rats that received PTK787 (**A**). Signal intensity (SI) changes at the peripheral and central parts of the tumors in PTK787 (**B**) and vehicle (**C**) treated animals show remarkable differences in the SI-change patterns. SI changes at the peripheral part (▴) in PTK787 treated tumor are significantly higher from that of central part (▪) of the PTK787 treated tumors as early as 3 minutes after the injection of contrast agent, which is obviously different from that of vehicle treated tumors. Analysis of K^trans^ values (**D**) shows significantly higher K^trans^ at the peripheral part of PTK87 treated tumors, compared to that of the corresponding central part. Vehicle treated tumors did not show any differences in K^trans^ values between the peripheral and central parts. Data are expressed as mean ± SEM, n≥3, * p<0.05.

**Figure 2:**
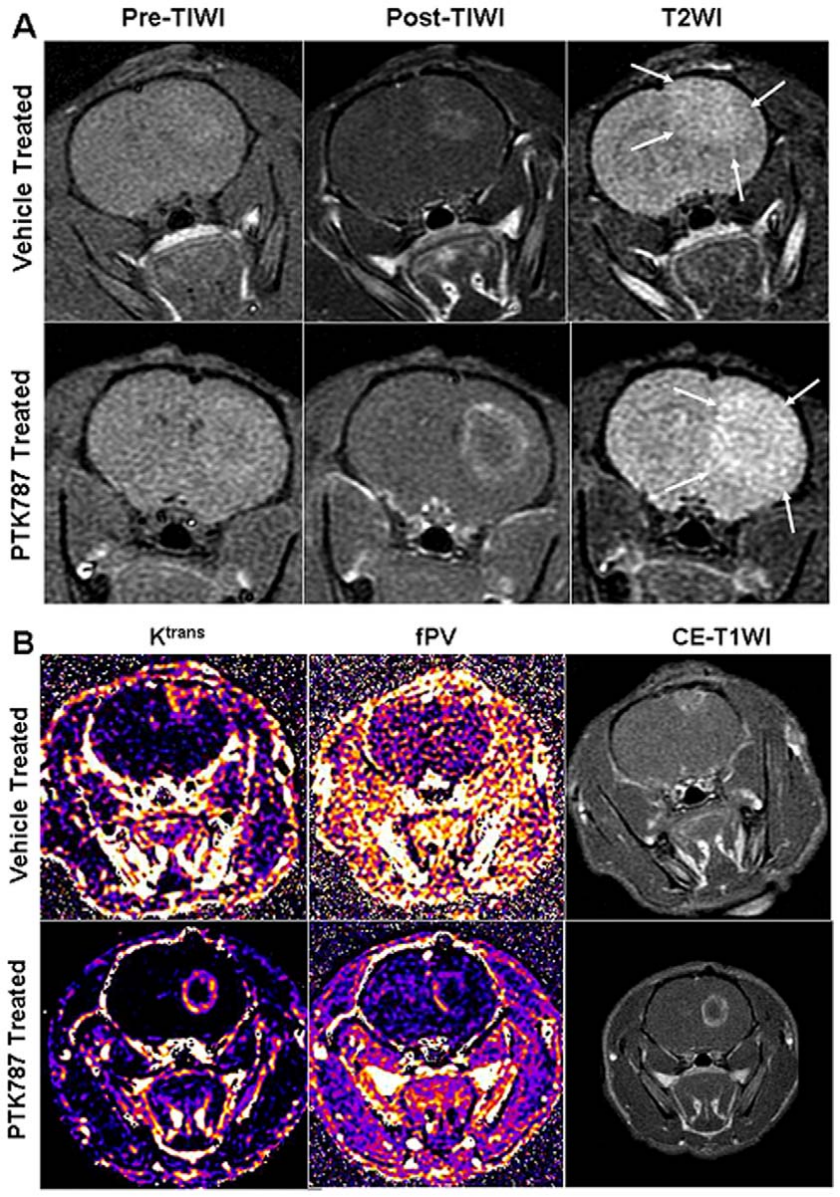
MRI and parametric analysis of PTK787 treated and vehicle treated tumors. (**A**) Representative cases from vehicle and PTK787 treated tumors show enhancement following administration of contrast agents (Post T1WI) and changes in T2WI. PTK787 treated tumor show larger tumor with prominent rim enhancement and central hollow compared to that of vehicle treated tumor. T2WI also show larger area of high signal intensity beyond the margin of enhancement (arrows). (**B**) Representative cases from vehicle and PTK787 treated tumors showing K^trans^, fractional plasma volume (fPV) and corresponding post contrast T1W images. Clearly increased K^trans^ and fPV at the periphery of PTK787 treated tumor (calculated K^trans^ and fPV maps are adjusted to same scale level for both vehicle treated and PTK787 treated tumors) indicate higher permeability and fPV, which are in agreement with the findings of dilated vessels at the peripheral part of PTK787 treated tumors (see below).

Vehicle and PTK787 treated rats were randomly selected from U-251 implanted tumors. Randomly selected animals from both groups also underwent pre treatment MRI to determine the growth of tumors. Pre-treatment tumor volumes were similar in both groups. However, significant differences (p = <0.001) were observed in the volume of the tumors in PTK787 treated group compared to that of vehicle treated group (**Figure 1A**). The changes were clearly visible on both, T2WI and post contrast T1WI (**Figure 2A**). High signal intensity areas seen on T2WI were larger in PTK787 treated tumors compared to that of vehicle treated tumors.

### DCE MRI

Signal intensity changes at the periphery and central parts of the PTK787 and vehicle treated tumors are shown in **Figure 1B and 1C**, respectively. In the PTK787 treated tumors (**Figure 1B** **)**, compared to that of vehicle treated tumors, higher signal intensity was observed at the peripheral part of the tumors as early as 3 minutes post contrast administration and the differences in signal intensity remained to the end of the dynamic MRI acquisition. On the other hand, the signal intensity at the central part of the PTK787 treated tumors did not change from the base line and this signal intensity was significantly different (p = <0.05) from that of peripheral part of the tumors as early as 3 minutes post contrast administration. The similar signal intensity differences between peripheral and central parts of the tumors were not observed in the vehicle treated groups (**Figure 1C**), where the signal intensity changes from the baseline were symmetrical for both, peripheral and central parts of tumors.

### Kinetic Analysis of DCE MRI

**Figure 1D** shows the K^trans^ values in PTK787 and vehicle treated tumors, and in contra lateral brains. Significantly higher (p = <0.05) K^trans^ values were observed at the peripheral part, compared to that of the central part and the contra lateral brain of the PTK787 treated tumors. However, similar significant differences between the peripheral and central parts were not observed in the vehicle treated animals. When we compared the K^trans^ values at the peripheral part of the two groups of tumors, higher K^trans^ values were observed in the PTK787 treated tumors, although significant difference was not detected (p = 0.14). In addition, higher fPV was observed at the peripheral part of the PTK787 treated tumors compared to that of the vehicle treated tumors (**Figure 2B**). Post contrast T1WI also supported the K^trans^ maps' findings where PTK787 treated tumors showed rim enhancement at the peripheral part of the tumors with central hollow (**Figure 2A, B**). **Figure 2B** also shows the representative maps of K^trans^ and fPV.

### *In Vivo* Expression of VEGFRs

**Figure 3:**
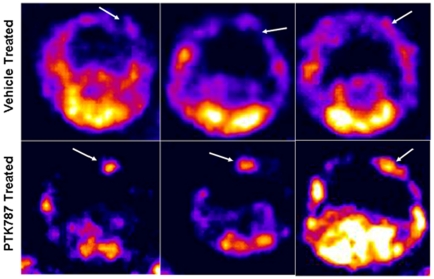
SPECT analysis of *in vivo* accumulation of Tc-99m-HYNIC-VEGF-c. VEGF-c (which targets both VEGFR2 and VEGFR3) was tagged with HYNIC chelators and then labeled with Tc-99m-pertechnetate (Tc-99m) and injected intravenously in PTK787 and vehicle treated rats. One hour after injection, SPECT images were obtained using dedicated animal scanner. All PTK787 treated rats showed increased accumulation of Tc-99m-HYNIC-VEGF-c in the tumors (lower panel, arrows) compared to that of vehicle treated tumors (upper panel, arrows).

To determine whether there were any changes in the expression of VEGFRs in the PTK787 and vehicle treated tumors, animals underwent SPECT scanning with Tc-99m tagged VEGF-c. All the animals that were treated with PTK787 showed higher activity of Tc-99-VEGF-c in the tumors (**Figure 3**) compared to that of the animals that were treated with vehicle. The higher expression of VEGFRs was also confirmed by western blotting and immunohistochemical studies (see below).

### Immunohistochemistry and Vascular Morphology

**Figure 4:**
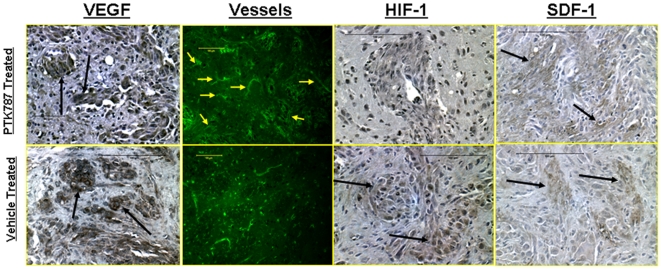
Immunohistochemistry of PTK787 and vehicle treated tumor showing expression of VEGF, HIF-1α, SDF-1 and vessel morphology. Expression of vascular endothelial growth factor (VEGF) (dark brown colored) at different parts of the PTK787 treated and vehicle treated tumors. There were no differences observed in the expression of VEGF on immunohistochemistry at different parts of the tumors treated with either PTK787 or vehicle. However, delineation of vessels using FITC tagged tomato lectin indicated higher number of dilated vessels at the tumor periphery in rats that received PTK787 treatment. These dilated vessels may be indicative of increased permeability, fPV and signal intensity changes in PTK787 treated tumors (see **Figures 1** **and** **2**). Increased permeability may also be due to increased VEGF expression. HIF-1α expression was mostly seen in the central part of the vehicle treated tumors (arrows), however, SDF-1 expressions were observed both in PTK787 and vehicle treated tumors (arrows).

**Figure 5:**
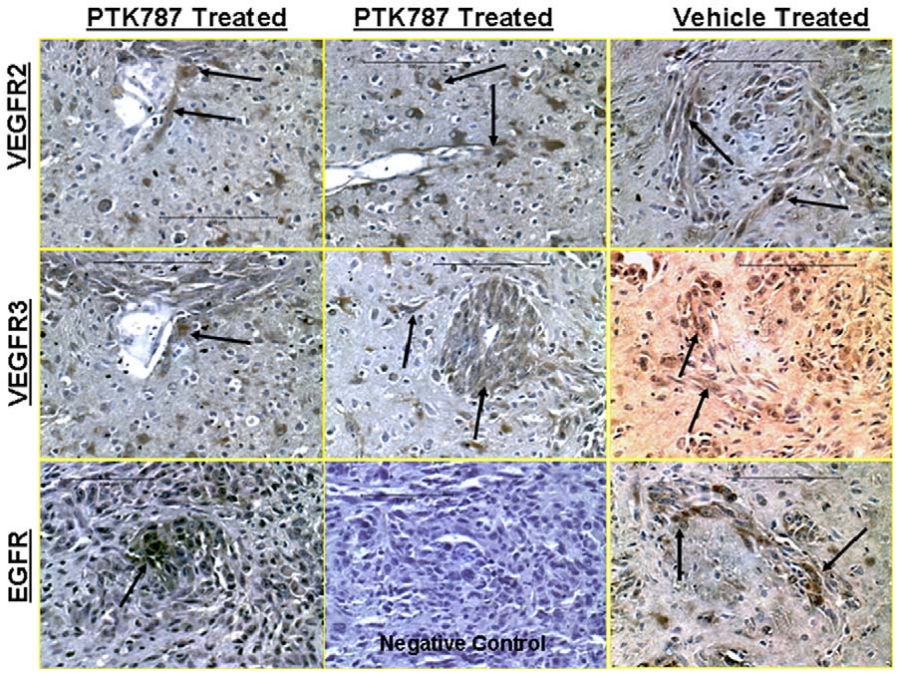
Immunohistochemistry of PTK787 and vehicle treated tumor showing expression of VEGFR2, VEGFR3 and EGFR. Immunohistochemistry confirmed the findings of SPECT studies, where PTK787 treated tumors showed increased expression of VEGFR2 and VEFGR3 at the peripheral parts of the tumors, especially around the vessels (arrows) compared to that of vehicle treated tumors (right column). Both PTK787 (lower panel, left column) and vehicle treated (lower panel, right column) showed expression of EGFR in the tumors. Lower panel, middle column show no brown cells in negative control slide.

There were no signs of necrosis within the tumor in both, PTK787 and vehicle treated tumors. **Figure 4** shows the expression of HIF-1α, SDF-1, VEGF and vascular morphology and density as detected by FITC-labeled tomato lectin. Numerous HIF-1α positive cells were observed in the central part of the vehicle treated tumors, whereas none to very few positive cells were observed in the central part of the PTK-787 treated tumors. Despite the differences in HIF-1α expression, both PTK787 and vehicle treated tumors showed the expression of SDF-1, especially at the peripheral parts. Both groups of tumors exhibited strong expression of VEGF in the central tumor mass as well as at the satellite tumor foci. FITC-labeled tomato lectin staining showed multiple dilated vessels at the peripheral part of the PTK787 treated tumors. Both, the numbers and the morphology of the vessels seen in PTK787 treated tumors were different from the vessels that were observed in vehicle treated tumors. PTK787 treated tumors showed high level of expression of VRGFR2 and VEGFR3 receptors both, in the lining of dilated vessels and in the tumor cells (**Figure 5**). Multiple cells in the brain (possibly microglia) near the tumors also exhibited high expression of VEGFR2 and VEGFR3. Although both receptors were also expressed in the vehicle treated tumors, the number of cells expressing the receptors in the surrounding brain was less compared to that of PTK787 treated tumors. Both vehicle and PTK787 treated tumors showed EGFR positive cells in the tumors.

### Western Blotting

**Figure 6:**
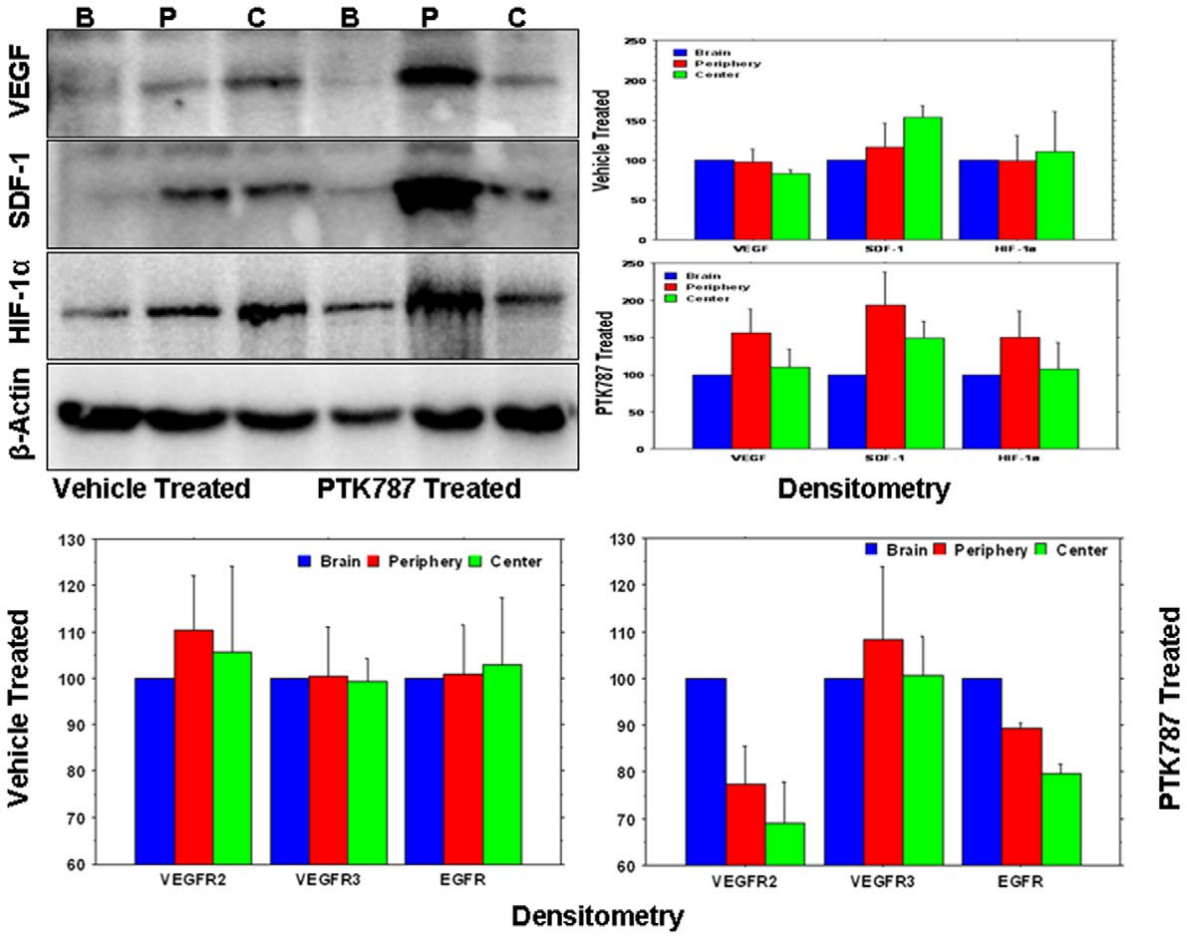
Western blot images and densitometry analysis of western blots. (**A**) Expression of different angiogenic factors (left panel) in the vehicle- and PTK787 treated tumors from representative cases at the peripheral (P), central part of the tumors (C) and contralateral brains (B). Note the increased expression of VEGF, SDF-1 and HIF-1α at the peripheral part of PTK787 treated tumors. Right panel shows the densitometry analysis of the blot (normalized to β-actin and contralateral brain). The analysis also confirmed the finding of the blot. Note the patterns of VEGF, SDF-1 and HIF-1α in PTK787 treated tumors which are different from that of vehicle treated tumors. (**B**) Densitometry analysis of VEGFR2, VEGFR3 and EGFR blot (normalized to β-actin and contralateral brain). Expression of VEGFR2, VEGFR3 and EGFR showed higher normalized values at the peripheral part of the PTK787 treated tumors compared to that of central part and the expression patterns are different in vehicle treated tumors. Please note that PTK787 treated tumors showed lower normalized values of VEGFR2 and EGFR both at the periphery and central parts of the tumors compared to that of corresponding contralateral brain; whereas vehicle treated tumors did not show any changes in the normalized values of VEGFR2 and EGFR compared to that of corresponding contralateral brain. Data are expressed as mean ± SEM, n = 3.

Higher expression levels of VEGF, SDF-1 and HIF-1α were observed at the peripheral part of the PTK787 treated tumors compared to that of central part (**Figure 6A**). Similar expression of the above mentioned angiogenic factors was also observed in the vehicle treated tumors. However, semi quantitative densitometry analysis of western blots, normalized to corresponding β-actin expression, and the expression of the same factors in contralateral brains showed different patterns of expression of VEGF, SDF-1 and HIF-1α in PTK787 and the vehicle treated tumors. All of the factors showed higher normalized values at the peripheral part of PTK787 treated tumors compared to that of central part and the similar patterns were not observed in the vehicle treated tumors. Similarly, expression of VEGFR2, VEGFR3 and EGFR showed higher normalized values at the peripheral part of the PTK787 treated tumors compared to the central part (**Figure 6B**) and the expression patterns were different in the vehicle treated tumors. It is to note that PTK787 treated tumors showed lower normalized values of VEGFR2 and EGFR both at the periphery and central parts of the tumors compared to that of corresponding contralateral brain; whereas vehicle treated tumors did not show any changes in the normalized values of VEGFR2 and EGFR compared to that of corresponding contralateral brain.

## Discussion

Results from these studies showed that orally administered PTK787 drug increased the tumor vascular permeability and tumor growth in rat model of human glioma. Surprisingly, even post contrast T1WI showed distinct differences between PTK787- and vehicle-treated tumors. The MR images showed bright contrast enhancement at the tumor rim and less contrast in the tumor center after therapy. T2WI also showed larger high signal intensity areas beyond the margin of post-contrast enhancement on T1WI. Analysis of the signal intensity ratio (tumor rims versus contra lateral brains) showed significantly higher values as early as 3 minutes after the administration of contrast agents. Our results demonstrated the unexpected, paradoxical effects of antiangiogenic treatment with VEGFRs inhibitors. Malignant gliomas are hypervascular tumors characterized by release of vascular endothelial growth factor (VEGF), an important regulator and promoter of angiogenesis [4]. Animal studies indicated that angiogenesis and increased vascular permeability are essential for the proliferation and survival of glioma cells [7]. Therefore, it was thought that anti-angiogenic therapy targeting VEGF or VEGF receptors (VEGFRs) would become an effective tool for controlling malignant glioma. However, our results are contradictory to this hypothesis and contradict the recent studies that reported decrease in tumor size of implanted glioma (and other tumors) as well as the decrease in permeability after treatment with VEGFRs inhibitor [25], [26]. However, in these reported studies, the investigators started the treatment soon after implantation and therefore might have not allowed for the sufficient time for the clinically relevant tumor angiogenesis to be generated. In our study, we started PTK787 treatment 7 days after tumor implantation, to mimic the clinical scenario of a developed malignant glioma with extensive angiogenesis. Recently, tyrosine kinase inhibitors (such as vetanalib) that target different VEGFRs have also been used in clinical trials [5], [6] with a limited success. However, it has been noted that continued anti-angiogenic therapy targeting only the VEGF-VEGFR system might activate pro-angiogenic factors other than VEGF, such as basic fibroblast growth factor (bFGF), stromal derived factor 1 (SDF-1) and Tie2 [5], and may mobilize circulating endothelial cells and bone marrow derived precursor cells that are known to promote angiogenesis [5], [16], [17]. Our results also showed increased expression of VEGF, HIF-1α, SDF-1 following the treatment with PTK787, indicating activation of pro-angiogenic factors, which may cause more angiogenesis and increase in vascular permeability. This effect is in part due to the accumulation of endothelial progenitor cells, since HIF-1α induced increase in SDF-1expression is a strong chemo-attractant for endothelial progenitor cells [27].

### Importance of DCE MRI in Measuring Vascular Permeability in Response to Therapy

DCE MRI has been used to validate the effect of anti-angiogenic [28], anti-vascular [29], radiation [30] and focused ultrasound therapies [31], to evaluate dose response [32] and to predict therapeutic responses in pre-clinical and clinical tests [33]. The results from many DCE MRI studies have been correlated to microvessel densities [34], tumor grades [35] and expression of VEGF [20]. There are only few reports on assessing response to PTK787 treatment of breast cancer or glioma models using DCE-MRI methods. Preliminary reports have shown that K^trans^ provides an early measure of response to therapy, with responding tumors showing a reduction in K^trans^ values. Non-responding tumors predominantly showed an increasing range of K^trans^ values; in well-perfused tissues this reflected changes in microvascular permeability. These techniques have also been employed in phase I/II clinical trials [32] of a VEGF inhibitor tyrosine kinase (PTK787), in which a reduction in K^trans^ value has been reported; however, investigators also noticed refractory cases following long-term use of antiangiogenic treatment [5]. From PTK787 antiangiogenic therapy point of view, we expected to observe a decrease in vascular permeability due to the interruption of receptors tyrosine kinase dependent pathway. However, our finding of an increase in K^trans^ under PTK787 treatment is contradictory and conflicts with the results reported by other groups [10], [12], [15]. In this study, DCE-MRI data showed a significant increase of K^trans^ values in U251 tumors treated with PTK787, but no change in the vehicle treated group. The increased values of K^trans^ correctly reflected the findings of histochemistry and western blot analysis, where vascular morphology and densities as well as the expression of VEGF and VEGF receptors were shown to be upregulated in PTK787 treated tumors. Our findings are in agreement with recently published data reporting an increase of permeability transfer constant under bevacizumab treatment [36]. Bevacizmab is a human-based antibody against VEGF. Our results demonstrate that blockade of VEGFRs signaling pathway may stimulate an alternative pathway for tumor growth and angiogenesis and the changes can be determined by DCE MRI.

### Activation of Alternate Pathways for Angiogenesis and the Importance of Our Findings for Future Treatment

Because of the hypervascular nature of glioblastoma and associated active angiogenesis, investigators have added anti-angiogenic treatment as an adjuvant to normalize the blood vessels and control the abnormal angiogenesis [3], [4], [5], [6]. Different targets have been selected to control abnormal angiogenesis. Bevacizumab, a humanised monoclonal antibody against VEGF is in clinical trial. Bevacizumab is commonly combined with cytotoxic chemotherapy and resulted in dramatic improvement on radiographic images, prolongation of progression free survival and less need for corticosteroids [4]. However, prolonged use of bevacizumab or cessation of anti-angiogenic treatment in glioma patients resulted in deteriorated clinical outcome [4], [6]. Different pro-angiogenic receptor tyrosine kinase inhibitors such as vetanalib, cediranib, sunitinib, etc have been used in clinical trails with varying degree of success [5], [37]. These receptor blockers were originally used for colorectal carcinoma and then later in glioma patients as adjuvants to the established treatments. Initial clinical trails showed remarkable improvement on MRI images with respect to tumor size and vascular permeability [5]. However, some reports showed extension of abnormal high signal intensity areas on T2-weighted images away from the tumor mass, which are thought to be due to invasive tumor mass (cells) [5]. Prolonged treatment with these receptor blockers also negatively impacted the outcome of the treatment. The possible mechanisms of the failure of these anti-angiogenic treatments are the activation of alternative angiogenesis pathways involved in expression of bFGF, SDF-1 and VEGF and increased invasiveness of the tumor cells [5], [16], [17]. Thus, the inhibitory therapy targeting VEGF and/or VEGFRs may end up in paradoxically enhancing angiogenic and pro-growth responses. In addition, as reported by our group and other investigators, SDF-1 is one of the potent chemo attractants for bone marrow derived endothelial progenitor cells due to the presence of CXCR4 receptors in these cells [27], [38], and it may be involved in enhanced angiogenesis and invasiveness of the tumor following treatment with VEGFRs inhibitors. Moreover, we have also reported the role of inflammatory cytokine RANTES, which also act as chemo attractant for these cells [39]. Any therapy would cause enhanced inflammation in the tumor sites that in turn may invite more endothelial progenitor cells and possibly increase angiogenesis. Our current results showing increased vascularity and permeability may support the hypothesis that increased accumulation of endothelial progenitor cells play a role in tumor angiogenesis. The increased expression of VEGF, SDF-1 and HIF-1α at the peripheral part of the PTK-787 treated tumors must have influenced permeability, dilatation of vessels (or increased angiogenesis) and increased the invasiveness of the tumors. Therefore, it is important to gain insight into the possible mechanisms that are activated during anti-angiogenic treatment to understand and potentially prevent its failure by using agents that would not only block the angiogenic factor receptors but also prevent the activation of alternative angiogenic pathways and release of other angiogenic factors.

### Importance of SPECT Studies in Delineating the Activation of Alternate Pathways and Monitoring the Treatment Progress

Investigators are now capable of easily tagging both, single photon emission computed tomography (SPECT) and positron emission tomography (PET) radioisotopes with peptides, amino acids or proteins [40]. Although image resolution of SPECT is not comparable to MRI and there is a need for anatomical images to localize the exact site of radioactivity, the sensitivity of SPECT is much superior to high resolution cellular MRI. The nuclear medicine imaging technique would be much more suitable for detecting expression of receptors in the tumor or receptor/ligand interaction using the tagging of radioactive isotopes to the ligands. There have been reports of making Tc-99m based SPECT agents for determining the expression of different angiogenic factors in the tumors [22], [23], [24], [40]. However, most of the studies have been performed in subcutaneous tumor models [24] and there has been no study on determining the expression of angiogenic factors/receptors in glioma following anti-angiogenic treatment utilizing SPECT analysis. We have successfully tagged Tc-99m with different proteins and peptides using the new generation chelator, HYNIC (Hydrazine Nicotinamide). Nicotinyl-containing HYNIC derivatives have been shown to be effective in tagging Tc-99m with annexin V, single chain VEGF, EGF and others [23], [24], [40]. Our results clearly showed the advantage of using SPECT method in detecting higher expression of VEGFRs in the PTK787 treated tumors, which are in agreement with the western blot findings. Despite the presence of VEGFR2 in the normal brain we could not detect any activity of Tc-99m-HYNIC-VEGF in the normal brain, which is due to intact blood brain barrier (BBB). However, the increased uptake of Tc-99m-HYNIC-VEGF in the PTK787 treated tumors might be due to active extravasation (due to BBB disruption and leakiness of dilated vessels as shown by tomato lectin staining) and it's attachment to the receptors expressed in the tumor cells and neovessels. Despite the loss of BBB in vehicle treated tumors (indicated by post contrast T1WI on MRI), Tc-99m tagged VEGF did not show increased activity in the tumors, which might indicate less neo-angiogenesis and lower amount of VEGFRs compared to that of PTK787 treated tumors.

### Clinical Implication

The results of these studies may have direct impact on the current clinical trials as well as the future anti-angiogenic therapy strategy, not only for glioma but for any other hypervascular tumors. Instead of relying only on MRI modality to follow up the patients, investigators may have a better alternative, the use of nuclear medicine techniques (both SPECT and PET) in determining the expression of various pro-angiogenic receptors during anti-angiogenic therapy as well as the ability to change the therapeutic strategy according to the findings. Further new therapies that serve as an improvement or even as an alternative to present treatments are needed. A newer treatment strategy should be designed to target not only the pro-angiogenic receptors but also the sources and the effects of angiogenic factors.

### Conclusion

Dynamic contrast-enhanced MRI (DCE-MRI) using a high molecular weight (MW) contrast agent (albumin-(GdDTPA)) showed significantly increased K^trans^ at the rims of the treated tumors compared to that of the central part of the treated as well as untreated (vehicle treated) tumors. Size of the tumors was also increased in the treated group. The expression of VEGFR2 detected by Tc-99m-HYNIC-VEGF SPECT was also significantly increased in the treated tumors. In PTK787 treated tumors, histological staining revealed increase in microvessel density in the close proximity to the tumor margin, while similar increased vascularity was not observed in vehicle treated tumors. Western blot analysis indicated increased expression of VEGF, SDF-1 and HIF-1α at the peripheral part of the treated tumors, compared to that of vehicle-treated and central part of the treated tumors. These findings indicate that PTK treatment may induced the over-expression of VEGF as well as the Flk-1/VEGFR2 receptor tyrosine kinase, especially at the rim of the tumor as proven by DCE-MRI, SPECT imaging and immunohistochemistry.

## Materials and Methods

### Ethics Statement

All animal experiments were performed according to NIH guideline and the protocol was approved by institutional animal care and user committee (IACUC) of Henry Ford Health System.

### Animal Model

Athymic nude rat 6–8 weeks of age and 150–170g of weight (Charles River Laboratory, Inc.) was anesthetized by intraperitoneal injection using ketamin/xylazine mixture (100 mg/kg ketamine, 15 mg/kg xylazine) and was placed on a stereotactic head holder. The surgical zone was shaved and swabbed with betadine solution, the eyes coated with Lacri-lube and the animal was immobilized in a small animal stereotactic device (Kopf, Cayunga, CA). After draping, a 1-cm incision was made 2 mm to the right of the midline, 1 mm retro-orbitally and the skull was exposed with cotton-tip applicators. A HP-4 dental drill bit was used with a micromanipulator to drill a hole 3 mm to the right and 1 mm anterior to the bregma, with the care not to penetrate the dura. A #2701 10 µL Hamilton syringe with a #4 point, 26s gauge-needle containing tumor cells (4×10^5^) in 5µl was lowered to the depth of 3.5 mm, then raised to the depth of 2.5 mm. The U251 cells were injected stepwise at a rate of 0.5µL/30 sec until the entire volume had been injected. During and after the injection, careful note was made of any reflux from the injection site. Two to three minutes after completing the injection the syringe was withdrawn in a stepwise manner. The surgical hole was sealed with a bone wax. Finally, the skull was swabbed with betadine before suturing the skin over the injection site. Total 44 animals were included in this study.

### Treatment with PTK787

Animals were randomly assigned to either the drug treatment (n = 24) or the control group (vehicle treated, n = 20). The control group was fed by gavage with solvent and the drug group was treated, also by gavage, with PTK787 tyrosine kinase inhibitor (a generous gift from Novartis, Basel, Switzerland). PTK787 was prepared for oral administration as follows: PTK787 was suspended in distilled water containing 5% of DMSO and 15% of sucrose. PTK was administered orally by gavage, once a day at a dose of 50 mg/kg per feeding. Drug administration started 7 days after tumor implantation and continued for five days in a row. Then, after the two days interval, drug administration was continued for another five days. On 22nd day, analyses were performed as follows: 1) group of animals underwent *in vivo* MRI, 2) group of animals underwent SPECT scanning with Tc-99m-HYNIC VEGF- and 3) group of animals were euthanized to collect non-perfused brain including tumors for western blotting study.

### *In Vivo* MR Imaging and Image Analysis

#### MR contrast media

Albumin-(GdDTPA) is a prototype of a water-soluble macromolecular contrast medium with 6 nm hydrodynamic diameter, synthesized in our laboratory following the method by Ogan et al. [41]. Albumin-(GdDTPA) was injected during imaging at a dose of 0.03 mmol Gd/kg. Although Albumin-(GdDTPA) is not a candidate for clinical application due to the slow and incomplete clearance and potential induction of immunologic toxicity, it has been used extensively in many experimental tumor models [42], [43], [44]. Blood volume and microvascular permeability were previously quantitatively estimated using kinetic models and correlated with histological analysis of capillary density [42], [45], [46].

#### Image acquisition

Rats were studied by MRI 22 days after implanting the tumor cells (U251). Rats were anesthetized with 2.0% isoflurane in oxygen carrier gas and secured to a customized cradle. A 26g dental catheter was inserted into a tail vein to facilitate the injection of contrast agents and while rats were in the MRI magnet the body temperature was maintained at 37.0°C. MR images were obtained with a 3.0 Tesla clinical system (Signa Excite, GE health) using 50 mm diameter×108 mm RF rung length small animal imaging coil (Litzcage small animal imaging system, Doty Scientific Inc, Columbia, SC). Pre- and post-contrast T1-weighted (T1W), T2-weighted (T2W), and three dimensional spoiled gradient echo (3D SPGR) images of the tumor bearing brain were acquired before and sequentially for 20 minutes after intravenous injection of Albumin-(GdDTPA). The three-dimensional field of view (FOV) of the image covered the entire brain. The following parameters were used to acquire the images. For T1W-image; TR = 625 ms, TE = 15 using a 160×128 matrix, FOV = 35 mm, and NEX = 4, effective slice thickness was 1 mm and 15 slices were imaged. For multiecho T2WI; TR = 2100 ms, TE = 15, 30, 45 and 60 using a 160×128 matrix, FOV = 35 mm, and NEX = 2. Effective slice thickness was 1 mm and 15 slices were imaged. To generate T1 map from precontrast images, 3D SPGR images with multiple flip angles 2°, 4°, 8°, 12°, and 15° were acquired. For dynamic post contrast 3D SPGR, a fixed flip angle of 15° was used. The following parameters were used to acquire the 3D SPGR images; TR = 11.3 ms, TE = 1.8 ms using a 128×128 matrix, FOV = 40×40×40 mm^3^, and NEX = 1. Effective slice thickness was 1 mm.

#### Kinetic analysis

We used the dynamic contrast-enhanced MR images and the following Patlak model to estimate the K^trans^ and the cerebral fPV (*V_p_*) maps [47], [48].
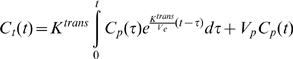
(1)where *V_e_* is the fractional volume of the extracellular volume and *C_t_*(*t*) and *C_p_*(*t*) are the tissue and the plasma concentrations over time, respectively. Dynamic contrast-enhanced MR imaging was performed using a transverse T1-weighted 3D SPGR acquisition that consisted of five precontrast and 51 dynamic post contrast images obtained up to 20 minutes after contrast injection. The K^trans^ and *V_p_* maps were generated by fitting the Patlak model in Eq. (1) to the gathered dynamic images using TOPPCAT software package [49], [50]. The K^trans^ and fPV maps at the periphery and the center of the tumors (both treated and control) were determined by drawing rectangular regions of interests (ROIs). The sizes of the ROIs were identical.

#### MR data analysis

To determine the changes in signal intensity at each time point following the administration of contrast agents, ROIs were drawn in the tumor periphery, tumor center and in the contralateral normal hemisphere. The sizes of the ROIs were identical for the three areas. The signal intensity changes were normalized to that of corresponding contralateral hemisphere using the following formula:

*signal intensity in the tumor (rim or center)/signal intensity in the contralateral hemisphere*

A dynamic curve indicating normalized signal intensity changes over time were created using Microsoft Office Excel 2003 software (Microsoft, CA). Tumor size was calculated from T1-weighted image sets obtained 24 min after Albumin-(GdDTPA) injection. Slices that showed enhanced areas were analyzed by defining a region of interest around the section of enhancement and measuring the area (cm^2^). The volume was calculated by multiplying by the slice thickness. This procedure was repeated for all slices showing enhancement and the areas were summed to determine a total tumor volume (cm^3^).

### SPECT Study

#### Rat VEGF-c

Rat recombinant VEGF-C was purchased from Prospec (Israel). VEGF-C, also known as Vascular Endothelial Growth Factor Related Protein (VRP), is a recently discovered member of VEGF growth factor family that is most closely related to VEGF-D. Rat VEGF-C cDNA encodes a pre-pro-protein of 416 amino acids residues and it is almost identical to mouse VEGF-C protein. Similar to VEGF-D, VEGF-C has a VEGF homology domain spanning the middle third of the precursor molecule and long N- and C-terminal extensions. Recombinant rat VEGF-C, lacking the N- and C-terminal extensions and containing only the middle VEGF homology domain, forms primarily non-covalently linked dimers. This protein is a ligand for both VEGFR-2/KDR and VEGFR-3/FLT -4.

#### Preparation of Hydrazine Nicotinamide (HYNIC)

Succinimidyl 6-hydrazinopyridine-3-carboxylate hydrochloride was synthesized and conjugated with rat VEGF-c as previously described [51]. The protein conjugate was purified with a Centricon C-3 diafilter with a 3,000 molecular weight cutoff. Then, the nicotinyl hydrazine conjugate of VEGF was radio labeled with ^99m^Tc-pertechnetate in the presence of tricine and stannous chloride as reported by Blankenberg et al. [22]

#### Image acquisition

An appropriate state of anesthesia was obtained using ketamine/xylazine 100/15 mg/kg. One hour after IV injection of 1 mCi Tc-99m-HYNIC-VEGF-c, SPECT images were obtained using a modified PRIZM 3000 gamma camera dedicated to animal studies and fitted with multi pinhole collimators (Bioscan, DC). The following image parameters were used: 256×256 matrix, 360° rotations, 10 projections, 180 sec per projection and 4×9 cm FOV. Total SPECT image acquisition time was 31 minutes. After the SPECT analysis animals were euthanatized and different tissues were collected for further analysis. The projection images were reconstructed with HiSPECT software (Bioscan, DC).

### Western Blotting

Animals used for western blot analysis were euthanized and fresh brains were collected (without perfusion), the tumor as well as contralateral brain were separated and snapped frozen. The collected frozen tumors were cut into peripheral and central portions, as described in our previous publication [27]. Tissues from the contralateral brain and the tumors were mechanically pulverized over dry ice and total protein (30–80 mg of the frozen tissue powder per sample) was extracted using ProteoExtract® Complete Mammalian Proteome Extraction Kit (Calbiochem®,USA) according to the manufacturer's instructions. Protein concentration in recovered protein extracts was determined using BioRad Protein Assay Dye Reagent Concentrate (Bio-Rad Laboratories) using Bovine Serum Albumin (BSA) as a standard. Forty microgram of total protein were resolved by electrophoresis using 8–12% SDS polyacrylamide gels, transferred to the nitrocellulose membrane (BioRad) and probed with specific primary antibodies: anti–VEGF (Santa Cruz Biotechnology, USA), anti-VEGFR2 (Upstate Biotech, USA), anti-VEGFR3 (Millipore, USA) anti-EGFR (Santa Cruz Biotechnology, USA), anti-SDF-1 (Santa Cruz Biotechnology, USA) and anti-HIF-1α (Lab Vision, USA). Primary antibody binding was detected by incubating the membrane in the presence of horseradish peroxidase (HRP) conjugated secondary polyclonal goat antibody and the signal was detected by enhanced chemiluminescence using the ECL Detection Kit (Amersham Biosciences). Density of the western blot images were normalized to corresponding β-actin and contralateral brain. The rectangular region of interest (ROI) was kept identical for all measurements. The following formula was used to normalization: ((density value of the band of interest/corresponding density value of β-actin)/density value of the corresponding band in contralateral brain)*100).

### Histopathology

Animals used for histochemical analysis were euthanized, perfused by intracardiac injection of 100 ml PBS followed by 3% paraformaldehyde and brains were removed and fixed in 3% paraformaldehyde containing 3% sucrose. Tissue sections were prepared from either frozen or paraffin tissue preparations. Standard histochemical staining procedures were performed as recommended by the suppliers of primary antibodies. The following antibodies were used to delineate the expression of corresponding antigens; anti-VEGFR2 antibody (Thermoscientific), anti-EGFR antibody (Santa Cruz Biotechnology, USA), anti-VEGFR3 antibody (Millipore, USA), anti-VEGF antibody (Thermoscientific), anti-SDF-1 antibody (Santa Cruz Biotechnology, USA) and anti-HIF1α antibody (Lab Vision, USA) To delineate the endothelial lining FITC labeled tomato lectin (Vectashield) was used.

#### Statistical analysis

All data are expressed as mean ± standard error of mean (SEM). Comparison between drug and vehicle treated groups was done by Student's t-test. Any p-value of <0.05 was considered significant.
